# Molecular control of nitric oxide synthesis through eNOS and caveolin-1 interaction regulates osteogenic differentiation of adipose-derived stem cells by modulation of Wnt/β-catenin signaling

**DOI:** 10.1186/s13287-016-0442-9

**Published:** 2016-12-07

**Authors:** Nadeeka Bandara, Saliya Gurusinghe, Shiang Yong Lim, Haying Chen, Shuangfeng Chen, Dawei Wang, Bryan Hilbert, Le-Xin Wang, Padraig Strappe

**Affiliations:** 1 School of Biomedical Sciences, Charles Sturt University, Wagga Wagga, NSW 2650 Australia; 2 O’Brien Institute Department, St. Vincent’s Institute of Medical Research, Fitzroy, VIC 3065 Australia; 3 Department of Surgery, St. Vincent’s Hospital, University of Melbourne, Melbourne, VIC 3002 Australia; 4 Department of Cardiology, Liaocheng People’s Hospital and Affiliated Liaocheng People’s Hospital of Shandong University, Liaocheng, Shandong 252000 China; 5 School of Animal and Veterinary Sciences, Charles Sturt University, Wagga Wagga, NSW 2650 Australia

## Abstract

**Background:**

Nitric oxide (NO) plays a role in a number of physiological processes including stem cell differentiation and osteogenesis. Endothelial nitric oxide synthase (eNOS), one of three NO-producing enzymes, is located in a close conformation with the caveolin-1 (CAV-1^WT^) membrane protein which is inhibitory to NO production. Modification of this interaction through mutation of the caveolin scaffold domain can increase NO release. In this study, we genetically modified equine adipose-derived stem cells (eASCs) with eNOS, CAV-1^WT^, and a CAV-1^F92A^ (CAV-1^WT^ mutant) and assessed NO-mediated osteogenic differentiation and the relationship with the Wnt signaling pathway.

**Methods:**

NO production was enhanced by lentiviral vector co-delivery of eNOS and CAV-1^F92A^ to eASCs, and osteogenesis and Wnt signaling was assessed by gene expression analysis and activity of a novel Runx2-GFP reporter. Cells were also exposed to a NO donor (NONOate) and the eNOS inhibitor, l-NAME.

**Results:**

NO production as measured by nitrite was significantly increased in eNOS and CAV-1^F92A^ transduced eASCs +(5.59 ± 0.22 μM) compared to eNOS alone (4.81 ± 0.59 μM) and un-transduced control cells (0.91 ± 0.23 μM) (*p* < 0.05). During osteogenic differentiation, higher NO correlated with increased calcium deposition, Runx2, and alkaline phosphatase (ALP) gene expression and the activity of a Runx2-eGFP reporter. Co-expression of eNOS and CAV-1^WT^ transgenes resulted in lower NO production. Canonical Wnt signaling pathway-associated Wnt3a and Wnt8a gene expressions were increased in eNOS-CAV-1^F92A^ cells undergoing osteogenesis whilst non-canonical Wnt5a was decreased and similar results were seen with NONOate treatment. Treatment of osteogenic cultures with 2 mM l-NAME resulted in reduced Runx2, ALP, and Wnt3a expressions, whilst Wnt5a expression was increased in eNOS-delivered cells. Co-transduction of eASCs with a Wnt pathway responsive lenti-TCF/LEF-dGFP reporter only showed activity in osteogenic cultures co-transduced with a doxycycline inducible eNOS. Lentiviral vector expression of canonical Wnt3a and non-canonical Wnt5a in eASCs was associated with induced and suppressed osteogenic differentiation, respectively, whilst treatment of eNOS-osteogenic cells with the Wnt inhibitor Dkk-1 significantly reduced expressions of Runx2 and ALP.

**Conclusions:**

This study identifies NO as a regulator of canonical Wnt/β-catenin signaling to promote osteogenesis in eASCs which may contribute to novel bone regeneration strategies.

**Electronic supplementary material:**

The online version of this article (doi:10.1186/s13287-016-0442-9) contains supplementary material, which is available to authorized users.

## Background

Mesenchymal stem cells (MSCs) have been isolated from various tissues such as adipose [1], heart [2], bone marrow [3, 4], and blood [5–9], and have the potential to differentiate into different lineages, including osteoblasts, chondrocytes, and adipocytes [10, 11]. The osteoblast differentiation program of MSCs is switched on by cell recruitment, and timely expression of genes including Runx2, alkaline phosphatase (ALP), type I collagen (ColA1), and osteocalcin (OC) followed by extracellular matrix mineralization [12]. This process can be induced by soluble molecules such as bone morphogenetic proteins (BMPs) [13] or Wnts [14–16] that activate several pathways and other various downstream signals such as protein kinase [17] and growth factors [18] to trigger osteoblast differentiation of mesenchymal stem cells.

Nitric oxide (NO) is a signaling molecule with a short half-life [19, 20]. It can react within the cell where it is produced or penetrate cell membranes to affect adjacent cells [21]. NO exerts a variety of physiological effects such as regulating blood pressure via smooth muscle relaxation [22], mediating immune responses [23], controlling cell proliferation [24], modulating apoptosis [20], promoting growth factor-induced angiogenesis [4, 21], accelerating wound healing [4, 25], and functioning as a neurotransmitter [26]. These responses can be mediated through activating the primary NO effector soluble guanylyl cyclase to produce cGMP [27] by NO-based chemical modifications of proteins through S-nitrosylation [28] or through epigenetic modification [29]. NO is known to play an important role in bone homeostasis. It is generated by many cell types present in the bone environment, most notably the osteoblast [30].

NO is synthesized from l-arginine by three isozymes of nitric oxide synthase (NOS), including neuronal NOS (nNOS), endothelial NOS (eNOS), and cytokine-inducible NOS (iNOS) [31]. Both iNOS [32, 33] and eNOS [34] have been shown to play a role in osteoblast differentiation. Mice lacking eNOS have shown marked bone abnormalities due to impaired osteoblast differentiation resulting in poor maintenance of bone mass [35, 36]. Gene expression data from neonatal calvarial osteoblasts from eNOS^–/–^ mice have shown downregulation of Runx2, Cbfa-1, and osteocalcin [37]. On the other hand, high concentrations of NO released due to the pathological iNOS expression promote bone resorption through induced osteoclastogenesis [38]. Therefore, an optimum level of NO is important to drive osteogenic differentiation of the MSCs.

In contrast with other NOS family members, eNOS is localized mainly in specific intracellular membrane domains, including the Golgi apparatus [39] and plasma membrane caveolae [40, 41]. A previously demonstrated direct interaction of eNOS with wild-type caveolin-1 (CAV-1^WT^) [42] has proposed that CAV-1^WT^ functions as an endogenous negative regulator of eNOS [43]. In this context, eNOS binds to the caveolin-1 scaffolding domain (CSD; amino acids 82–101) [44] and, furthermore, Thr-90 and Thr-91 (T90 and T91), and Phe-92 (F92) were identified as critical residues for eNOS binding and inhibition [41]. Genetic modification of endothelial cells through overexpression of a mutated version of CAV-1 with a phenylalanine to alanine substitution at the amino acid position 92 (CAV-1^F92A^) resulted in increased NO production, overcoming the inhibitory effect of CAV-1^WT^ [41].

In the present study, we tested the hypothesis that molecular control of NO synthesis in equine adipose-derived stem cells (eASCs), can promote osteogenic differentiation where endogenous eNOS is not available, by recreating the interaction between eNOS and CAV-1 (CAV-1^WT^ and CAV-1^F92A^) regulates the osteogenic differentiation of eASCs. Our results indicate that the optimum level of NO induces osteogenic differentiation through activation of the downstream canonical Wnt/β-catenin signaling pathway.

## Methods

### Cell culture

eASCs were isolated from subcutaneous adipose tissue as previously described [45]. All sampling was carried out using protocols approved by the Charles Sturt University Animal Care and Ethics Committee. Human embryonic kidney 293 T cells (HEK293T) (ATCC, VA, USA) and eASCs were cultured and maintained in Dulbecco’s modified Eagle’s medium (DMEM; Sigma-Aldrich, MO, USA) supplemented with 10% (v/v) fetal bovine serum (FBS; Bovogen, VIC, Australia), 100 U/mL penicillin, 100 μg/mL streptomycin, and 2 mM l-glutamine (Invitrogen) (growth medium) at 37 °C and 5% CO_2_.

### Plasmid constructs

The list of cDNA for the genes of interest used in this study is listed in Table 1, and was used for construction of lentiviral vectors. HF Phusion (New England Biolabs; NEB) DNA polymerase was used for all the polymerase chain reactions (PCRs) and all the restriction endonucleases were purchased from NEB unless indicated otherwise.

**Table 1:** Lentiviral vectors and reporter constructs used in this study

Lentiviral vector	Relevant properties	Source or reference
pWPT-eNOS	CMV-eNOS	This study
FUW-eNOS	TetO-eNOS	This study
pWPT-CAV-1^F92A^	CMV-CAV-1^F92A^	This study and [41]
pLVX-CAV-1^WT^	CMV-CAV-1^WT^	This study
pTRIP-Runx2.Hsp68-eGFP	Runx2.Hsp68-eGFP	This study and [46]
pLX304-Wnt3a	CMV-Wnt3a	DNASU (HsCD00436739)
pLX304-Wnt5a	CMV-Wnt5a	DNASU (HsCD00442542)
pRRL-TCF/LEF-GFP	TCF/LEF-dGFP	Addgene (#14715)

To construct the CMV promoter-driven eNOS expressing lentiviral vector, a codon optimized eNOS gene was synthesized [4] and subcloned into the pWPT-GFP lentiviral plasmid (Addgene, MA, USA) using *Bam*H1 and *Sal*1 restriction endonucleases. Doxycycline (DOX) inducible eNOS construct was prepared by amplifying the eNOS gene using the forward (ATCAGAATTCATGGGCAACCTGAA) and reverse primer (ATCAGAATTCTCATCAGGGGCTGT) by introducing *Eco*R1 restriction sites (underlined sequences in both forward and reverse primers) at both the 5’ and 3’ ends of the final PCR product, followed by subcloning the PCR product into FUW-TetO vector (Addgene). Human wild-type caveolin-1 (CAV-1^WT^) expressing lentiviral vector was constructed by inserting the full length CAV-1^WT^ (Addgene) into pLVX-AcGFP1-C1 and pLVX-DsRed-C1 (Clontech, CA, USA) using *Eco*R1 and *Bam*H1 restriction endonucleases. A mutated caveolin-1 (CAV-1^F92A^) in which phenylalanine (F) at the amino acid position 92 was replaced with alanine (A) [41] was synthesized (Geneart), amplified by PCR by introducing *Bam*H1 and *Sal*1 sites at the 5’ and 3’ ends of the PCR product, respectively, via forward primer (ATCAGGATCCATGTCTGGGGGCA) and reverse primer (ATCAGTCGACTTATATTTCTTTCTG) (restriction sites are underlined). The PCR product was then ligated into the pWPT-GFP lentiviral vector (Addgene) at the *Bam*H1 and *Sal*1 restriction sites replacing GFP. Wnt3a and Wnt5a expressing lentiviral plasmids were purchased from DNAsu plasmid repository.

### GFP reporter constructs

A 343‐bp fragment of the Runx2 enhancer region (sequence information was kindly provided by Toshihisa Komori at the Department of Cell Biology, Nagasaki University) [46] was synthesized together with the sequence of the Hsp68 minimal promoter (GenScript, NJ, USA). The entire fragment was then subcloned into pTRIP-eGFP lentiviral vector at the *Mlu*1 and *Bam*H1 restriction sites replacing an insulin-specific promoter, upstream of the enhanced GFP (eGFP) coding sequence. The Wnt responsive lentiviral TCF/LEF-dGFP reporter system was purchased from Addgene.

### Lentiviral vector production and transduction of equine adipose stem cells

Lentiviral vectors used in this study (Table 1) were generated by four plasmid transfection of HEK293T cells. Briefly, each well of a six-well tissue culture plate was coated with 50 μg/mL of dl-lysine (Sigma-Aldrich) in phosphate-buffered saline (PBS) and incubated for 2 h at 37 °C. HEK293T cells were then seeded at a density of 1 × 10^6^ cells per well, 24 h prior to transfection of 6.3 μg of packaging plasmid psPAX2 (Addgene), 3.1 μg of Rev expression plasmid pRSV Rev (Addgene), 3.5 μg of VSV-G envelop pMD2.G (Addgene), and 10 μg of the gene of interest expression transfer vector using a standard calcium phosphate transfection method [47]. Seventeen hours post-transfection, the media was changed and supernatant containing lentiviral vectors were collected at 48 h and 72 h post-transfection, combined, and filtered through a 0.45-μM PVDF filter, and used for eASC transduction in the presence of 4 μg/mL Polybrene (Sigma-Aldrich).

### Osteogenic differentiation

eASCs were seeded in a 12-well plate (11,000 cells/cm^2^) in triplicate in growth medium overnight followed by transduction with eNOS, CAV-1^WT^, and CAV-1^F92A^ lentiviruses. After 3 days, growth medium was replaced with osteogenic induction medium (OM; growth medium + 0.2 mM 2-phospho-l-ascorbic acid trisodium salt + 10 nM dexamethasone + 10 mM β-glycerol phosphate; Sigma-Aldrich). Non-induced control cells were cultured in growth medium. Medium was changed every 3 days (see Fig. 2a below).

After 11 days incubation in OM or control growth medium, cells were washed with PBS and fixed with 4% (w/v) paraformaldehyde (Sigma-Aldrich) for 20 min, washed with distilled water, and then stained with 2% (w/v) Alizarin Red S (pH 4.2) for 20 min. Stained cells were washed with distilled water prior to assessment by light microscopy using a Nikon Eclipse Ti-S inverted microscope (Nikon, Japan).

### Alizarin Red S quantification

Quantification of Alizarin Red S staining was performed as previously described [48]. Briefly, after staining the cells with Alizarin Red S for 20 min, 10% acetic acid was added to the 12-well cell culture plate and incubated for 30 min with shaking. The Alizarin Red S stain was extracted and the absorbance was measured at 405 nm in parallel with Alizarin Red S standards comprising of serial 1:2 dilutions of 50 mM Alizarin Red S (pH 4.2).

### Quantitative real-time PCR

Total RNA from transduced and control cells after 11 days of incubation in OM or growth medium was isolated using the PureZol reagent (Bio-Rad, CA, USA) according to the manufacturer’s instructions, and the concentration of isolated RNA was determined using a Nanodrop spectrophotometer (Thermo Fisher Scientific), treated with RQ1 RNase free DNase (1 U/1 μg RNA; Promega, WI, USA). cDNA was synthesized with 1 μg RNA from all samples using a High Capacity Reverse Transcription Kit (Thermo Fisher Scientific). Quantitative real-time PCR assays were performed on a BioRad CFX96 Real-Time system (Bio-Rad) using the SsoFast EvaGreen Supermix (Bio-Rad). Primer sequences used for target gene amplification are described in Table 2. Assays were performed in triplicate and target gene expression was normalized to equine β-actin mRNA levels using the ΔΔC_t_ method.

**Table 2:** Primers used for reverse transcription quantitative polymerase chain reaction

Gene	Forward (5’ > 3’)	Reverse (5’ > 3’)	Accession number
β-actin	ATGGATGATGATATCGCCGC	AGGTCTCAAACATGATCTGGG	NM_001081838
Runx2	TCCACCACGCCGCTGTCT	TCAGTGAGGGATGAAATGCT	XM_005603968
ALP	TCATCGACATCTGGAAGAGC	GCTCAAAGAGACCCAAGAGG	XM_008537803
Wnt3a	TCAAGATCAGCATCCAGGAG	GTTGACAGTGGTGCAGTTCC	XM_014730421
Wnt8a	CCCAAGGCCTATCTGACCTA	AGCCTGTTGTGAGTGGACAG	XM_014730656
Wnt5a	CGAAGACAGGCATCAAAGAA	TATCTGCATAACCCTGCCAA	XM_014731495
GFP	AGCACTGCACGCCGTAGGTC	CGAGCTGGACGGCGACGTAA	KX349734

### Immunocytochemistry and confocal microscopy

Immunocytochemical detection of eNOS and caveolin-1 (CAV-1^WT^ and CAV-1^F92A^) expression in eASCs was performed as follows. Briefly, cells were fixed in 4% paraformaldehyde for 20 min at 37 °C, treated with 0.1% Triton-X100 in PBS for 10 min, and blocked in a 10% FBS in PBS solution for 30 min at room temperature. This was followed by a 2-h incubation with a primary mouse monoclonal anti-eNOS antibody (BD Biosciences, CA, USA) or rabbit polyclonal anti-CAV-1 antibody (Cell Signaling Technology, MA, USA), and subsequently with an anti-mouse IgG secondary antibody conjugated with Alexa 488 (Cell Signaling Technology) or anti-rabbit IgG secondary antibody conjugated with Alexa 555 (Cell Signaling Technology) for 1 h and counterstained with DAPI for nuclear staining (Sigma-Aldrich). eNOS and CAV-1 co-localization was observed by confocal microscopy (Nikon).

To detect β-catenin expression, eNOS transduced cells (with or without DOX treatment) and un-transduced cells were fixed in 4% paraformaldehyde for 20 min at 37 °C, treated with 0.1% Triton-X100 in PBS for 10 min, and blocked in a 10% FBS in PBS solution for 30 min at room temperature. This was followed by an overnight incubation with a primary rabbit monoclonal anti-β-catenin antibody (Cell Signaling Technology) and subsequently with an anti-rabbit IgG secondary antibody conjugated with Alexa 488 (Cell Signaling Technology) for 1 h and counterstained with DAPI.

### GFP reporter assays

For GFP-based reporter assays for both TCF/LEF-dGFP and Runx2.Hsp68-eGFP, cells transduced with the TCF/LEF-dGFP and Runx2.Hsp68-eGFP were subjected for reverse transcription quantitative PCR (RT-qPCR) for GFP expression and fluorescence microscopic analysis, respectively.

### Nitric oxide detection

Extracellular NO production was measured using the Griess reagent (Promega) according to the manufacturer’s instructions and measurement of absorbance at 540 nm. Triplicates of each sample were measured at each time-point during osteogenic differentiation from day 0 to day 11.

### Statistical analysis

All experiments were performed in triplicate and at least three times. Data are presented as mean ± SEM. The statistical significances were determined by one-way analysis of variance (ANOVA) followed by Tukey’s test. All tests were performed using the statistical software GraphPad Prism 6 (GraphPad, CA, USA). *p* < 0.05 was considered statistically significant.

## Results

### eASC characterization

eASCs were spindle-shaped and adherent to plastic tissue culture dishes (data not shown). We have previously reported their tri-linage differentiation potentials [49].

### eNOS and caveolin-1 expression in eASCs

**Fig. 1:**
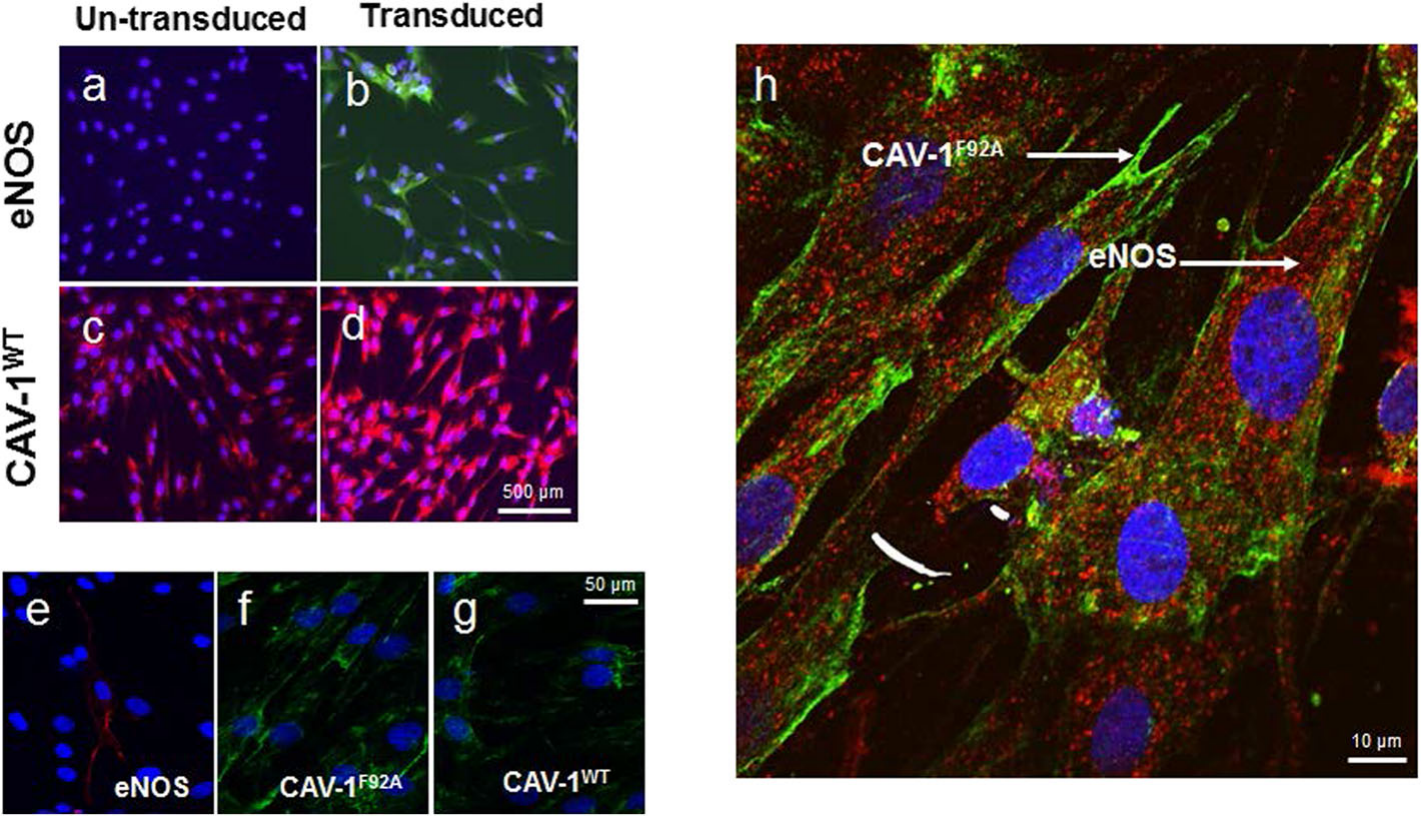
Immunofluorescence localization of eNOS, CAV-1^F92A^, and CAV-1^WT^ in lentiviral transduced eASCs and un-transduced cells. **a** Un-transduced eASCs show no endogenous endothelial nitric oxide synthase (*eNOS*) expression. **b** eNOS-transduced eASCs (*green*) show strong cytoplasmic expression. **c** Un-transduced eASCs show endogenous caveolin expression (*red*). **d** Wild-type caveolin-1 (*CAV-1* ^*WT*^)-transduced eASCs show significantly stronger expression. When **e** eNOS, **f** mutated caveolin-1 (*CAV-1* ^*F92A*^), and **g** CAV-1^WT^ were transduced to eASCs using lentiviral vectors, eNOS showed cytoplasm localization (*red*) whereas both the CAV-1^F92A^ and CAV-1^WT^ showed plasma membrane localization (*green*). **h** Confocal microscopy analysis of co-transduction of eASCs with eNOS and CAV-1^F92A^ resulted in cytoplasmic eNOS expression (*red*) and membrane localization of CAV-1^F92A^ (*green*)

eNOS activation is controlled at the cell plasma membrane significantly by CAV-1, a major structural protein in caveolae [50, 51]. We investigated eNOS and CAV-1 expression in un-transduced and transduced eASCs by immunofluorescence microscopy. Wild-type CAV-1 (CAV-1^WT^)-transduced eASCs (eASC^CAV-1WT^) and un-transduced eASCs (eASC^WT^) expressed CAV-1 protein; notably, the CAV-1 expression was increased in eASC^CAV-1WT^ (Fig. 1d) compared to eASC^WT^ (Fig. 1c). Interestingly, eNOS expression was absent in eASC^WT^ (Fig. 1a), whereas strong eNOS expression was observed in eNOS-transduced cells (eASC^eNOS^) (Fig. 1b). Next, we examined the localization of eNOS, CAV-1^WT^, and mutated CAV-1 (CAV-1^F92A^) in genetically modified eASC by confocal microscopy. As expected, eNOS expression was detected at the cytoplasm (Fig. 1e), whereas both the CAV-1^F92A^ (Fig. 1f) and CAV-1^WT^ (Fig. 1g) expressions were observed at the plasma membrane, confirming that F92A mutation of CAV-1 does not affect its cellular localization. Co-localization of eNOS and CAV-1^F92A^ in co-transduced eASCs with eNOS and CAV-1^F92A^ (eASC^eNOS+CAV-1F92A^) was examined by confocal microscopy with expression of eNOS in the cytoplasm and CAV-1^F92A^ at the plasma membrane (Fig. 1h). As controls for primary antibodies, immunostaining was carried out in the absence of primary antibodies specific to eNOS and CAV-1 (Additional file 1: Figure S1).

### NO enhances osteogenic differentiation

**Fig. 2:**
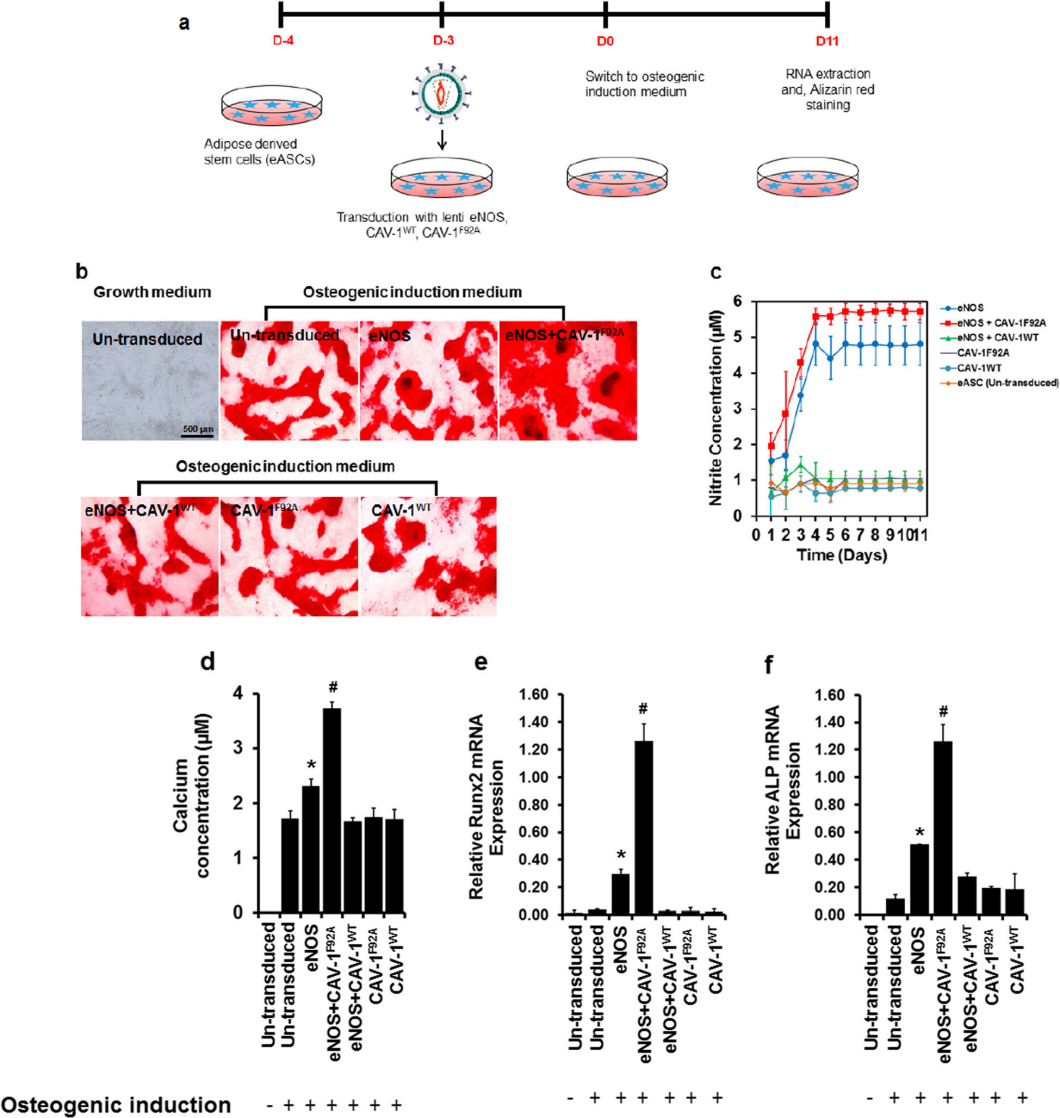
Nitric oxide promotes osteogenic differentiation of eASCs. **a** Schematic of lentiviral transduction and osteogenic induction strategy. **b** Alizarin Red S staining after 11 days in osteogenic induction medium or growth medium. **c** Nitric oxide release from cells undergoing osteogenic differentiation through quantification of nitrite by Greiss assay. **d** Quantification of calcium deposition. Relative mRNA transcripts analysis by qPCR of **e** Runx2 and **f** alkaline phosphtase (*ALP*) osteoblast markers showing increased expressions in endothelial nitric oxide synthase (*eNOS*) and eNOS + mutated caveolin-1 (*eNOS + CAV-1* ^*F92A*^) transduced cells compared to all other treatments. *p < 0.05, versus eNOS+CAV-1WT, CAV-1WT, CAV-1F92A, eASC (osteogenic induction), eASC (growth medium). ; ^#^ *p* < 0.05 versus eNOS, eNOS+CAV-1WT, CAV-1WT, CAV-1F92A, eASC (osteogenic induction), eASC (growth medium).*CAV-1* ^*WT*^ wild-type caveolin-1

To examine the role of the NO signaling in eASCs osteogenesis, we first compared the osteogenic differentiation between eNOS transduced (eASC^eNOS^) and un-transduced eASCs (eASC^WT^). A greater number of Alizarin Red S-positive nodules were induced in the eASC^eNOS^ cultures compared to eASC^WT^ culture after 11 days (Fig. 2b). NO synthesis was also significantly increased in eASC^eNOS^ compared to eASC^WT^ (Fig. 2c). Quantification of calcium deposition showed increased levels of calcium deposition in eASC^eNOS^ compared to eASC^WT^ (Fig. 2d). Quantitative analysis of Runx2 (Fig. 2e) and ALP (Fig. 2f) gene expression were also significantly upregulated in the eASC^eNOS^ cultures compared to the eASC^WT^ cultures.

**Fig. 3:**
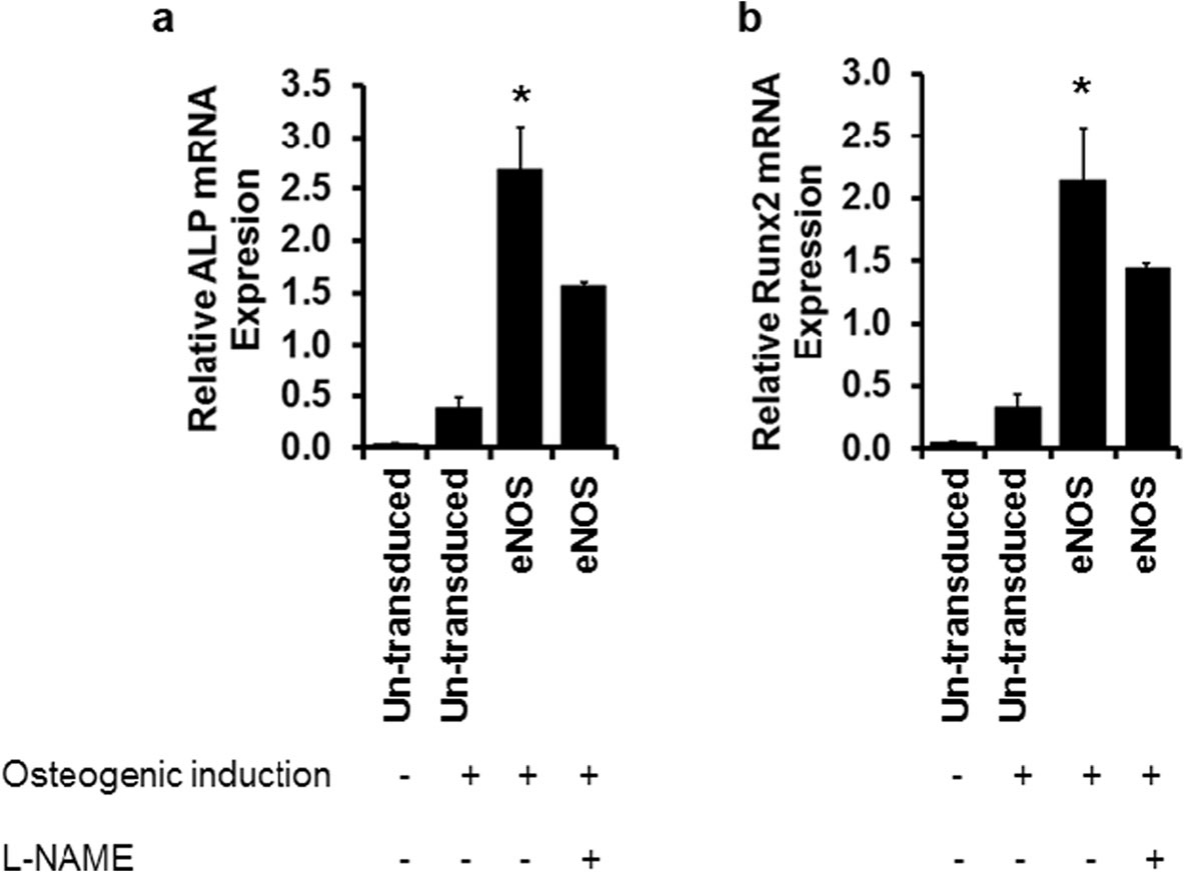
Inhibition of endothelial nitric oxide synthase (*eNOS*) through l-N^G^-nitroarginine methyl ester (*l* *-NAME*) treatment downregulates eASC osteogenic differentiation. Relative mRNA transcripts analysis by qPCR showing that 2 mM l-NAME treatment decreased the expression of osteoblast markers **a** alkaline phosphatase (*ALP*) and **b** Runx2 in eNOS transduced eASCs compared to untreated eNOS transduced cells. *p < 0.05, versus eNOS (l-NAME), eASC (osteogenic induction), and eASC (growth medium)

NO-mediated osteogenic differentiation was further highlighted by inhibition of eNOS activity. eASC^eNOS^ were treated with 2 mM of the nitric oxide synthase inhibitor, l-N^G^-nitroarginine methyl ester (l-NAME) for 11 days. l-NAME treatment resulted in a significant downregulation of osteoblast-specific marker expressions, ALP (Fig. 3a) and Runx2 (Fig. 3b), compared to untreated eASC^eNOS^.

### Co-expression of eNOS and CAV-1^F92A^ enhances NO production and osteogenic differentiation

Lentiviral vectors expressing eNOS and CAV-1^F92A^ (mutant) or CAV-1^WT^ (wild-type) were co-expressed in eASCs, eASC^eNOS+CAV-1F92A^, and eASC^eNOS+CAV-1WT^, respectively. Co-expression of eNOS and CAV-1^F92A^ promoted osteogenesis as evident by Alizarin Red S staining (Fig. 2b) and calcium deposition (Fig. 2d) compared to eNOS alone (eASC^eNOS^), and NO levels were also significantly increased in the eASC^eNOS+CAV-1F92A^ (Fig. 2c). Co-expression of eNOS with CAV-1^WT^ in eASCs (eASC^eNOS+CAV-1WT^) reduced NO production (Fig. 2c) and also osteogenesis as evident by Alizarin Red staining (Fig. 2b) and calcium deposition (Fig. 2d). Quantitative real-time PCR analysis revealed that Runx2 (Fig. 2e) and ALP (Fig. 2f) were significantly upregulated in the eASC^eNOS+CAVF92A^ cultures and downregulated in the eASC^eNOS+CAV-1WT^ cultures as compared with the eASC^eNOS^ cultures (Fig. 2e and f).

### Exogenous NO donor enhances osteogenesis of eASC in a dose-dependent manner

**Fig. 4:**
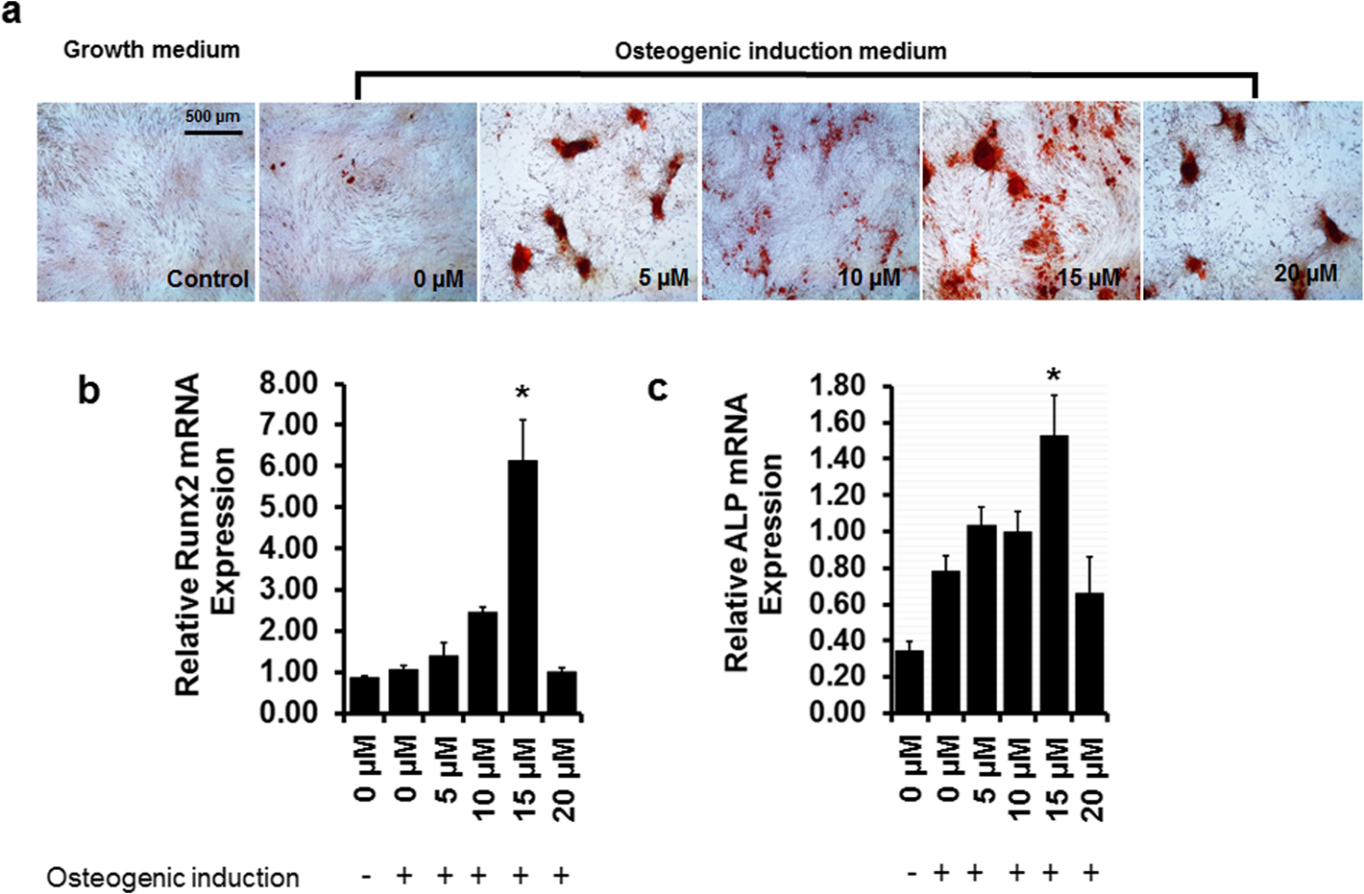
The exogenous nitric oxide donor NONOate promotes osteogenic differentiation in a dose-dependent manner. **a** Alizarin Red S staining of eASCs after 11 days in osteogenic induction medium or growth medium. Relative mRNA transcript analysis shows a significant increase of osteoblast markers **b** Runx2 and **c** alkaline phosphatase (*ALP*) when the eASCs were treated with 15 μM NONOate. *p < 0.05, versus 0 μM, 5 μM, 10 μM, and 20 μM NONOate, and control (growth medium)

To confirm the direct role of NO levels on eASC osteogenesis, we treated eASCs with a concentration range of exogenous NO donor (NONOate; Sigma-Aldrich). Treatment with exogenous NO donor promoted osteogenesis from 5 μM to 15 μM but this was reduced with high concentrations of NO donor (20 μM) as evident by Alizarin Red staining (Fig. 4a). NONOate treatment also resulted in a dose-dependent increase in Runx2 (Fig. 4b) and ALP (Fig. 4c) gene expression, in which maximum levels of both were achieved with 15 μM NONOate; notably, significantly lower levels of Runx2 and ALP expression were observed with 20 μM NO donor treatment.

### NO promotes endogenous Runx2 expression in differentiating eASCs

**Fig. 5:**
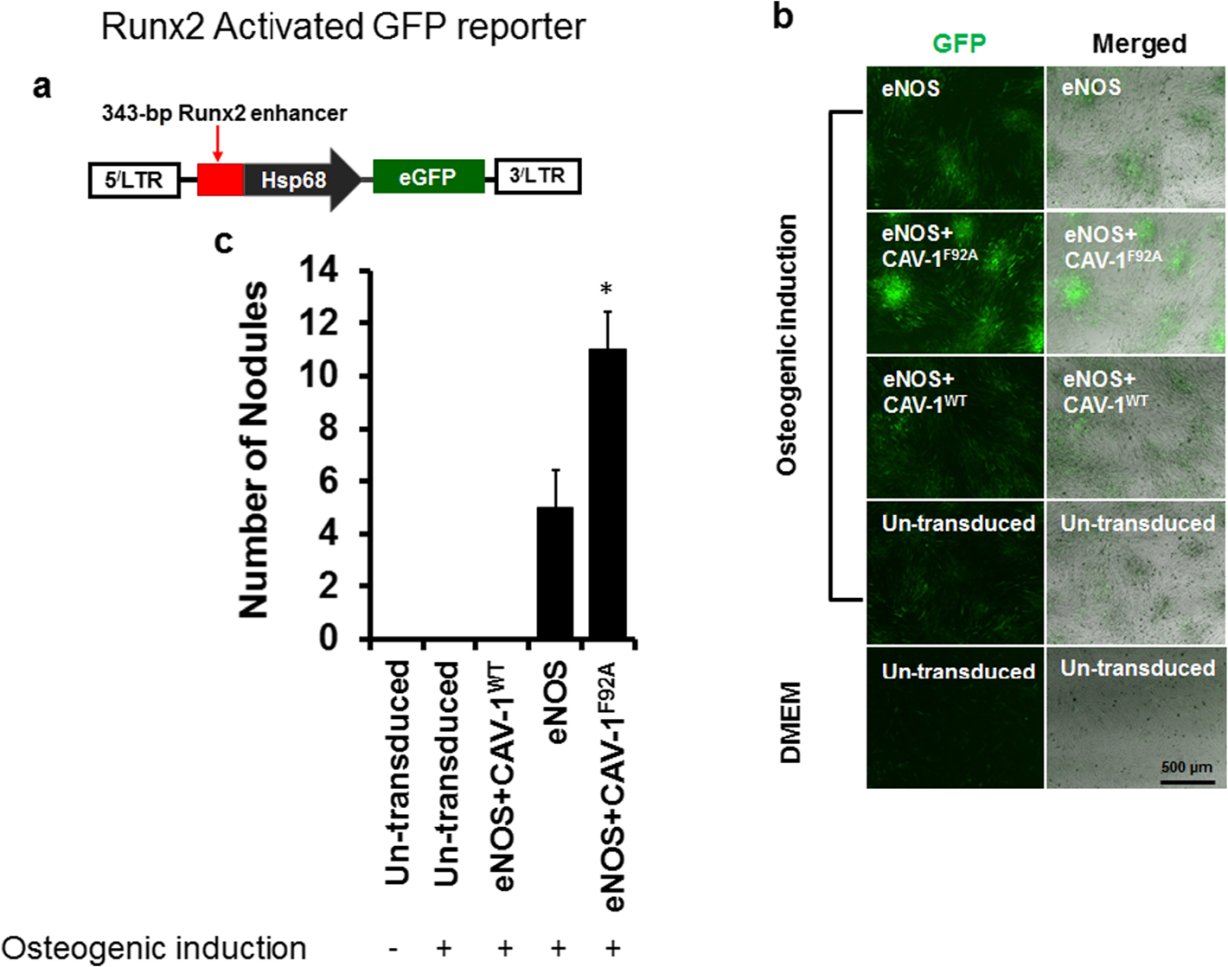
Activity of a Runx2 reporter during eASC osteogenic differentiation. **a** Schematic of the Runx2.Hsp68-eGFP lentiviral reporter used to transduce eASCs. The construct contains 343 bp of Runx2 enhancer and Hsp68 minimal promoter upstream to the enhanced green fluorescent protein (*eGFP*). **b** Runx2 reporter containing eASCs were transduced with endothelial nitric oxide synthase (*eNOS*), eNOS + mutated caveolin-1 (*eNOS + CAV-1* ^*F92A*^), and eNOS + wild-type caveolin-1 (*eNOS + CAV-1* ^*WT*^) following osteogenic induction up to 11 days, and GFP signals were detected by fluorescence microscopy and **c** increased numbers of osteogenic nodules were observed. *p < 0.05, versus eNOS, eNOS + CAV-1^WT^, eASC (osteogenic induction), and eASC (growth medium). *DMEM* Dulbecco’s modified Eagle’s medium

To monitor the effect of NO on endogenous Runx2 expression in differentiating eASCs, we generated a GFP lentiviral reporter system under the control of a Runx2 enhancer fused to the Hsp68 promoter (Runx2.Hsp68-eGFP; Fig. 5a) based on a novel Runx2 enhancer. eASCs which were stably transduced with the Runx2 reporter showed low levels of GFP expression in mostly undifferentiated cells (Fig. 5b), whereas GFP expression as a result of Runx2 promoter activity in differentiating eASCs was increased (Fig. 5b). When eASCs were transduced with eNOS (eASC^eNOS^), the GFP signals were increased compared to eASCs co-transduced with eNOS and CAV-1^WT^ (eASC^eNOS+CAV-1WT^) and un-transduced control (eASC^WT^) (Fig. 5b). Interestingly, we observed that endogenous Runx2 activity was remarkably increased when the eASCs were co-transduced with eNOS and CAV-1^F92A^ (eASC^eNOS+CAV-1F92A^) (Fig. 5b) compared to eASC^eNOS^, eASC^eNOS+CAV-1WT^, and eASC^WT^. Osteogenic nodule formation was significantly increased in the eASC^eNOS+CAV-1F92A^ (Fig. 5c), and these result suggests that endogenous Runx2 expression is less active in undifferentiated eASCs and its expression is significantly increased through NO signaling during osteogenic differentiation.

### NO modulates Wnt signaling to promote osteogenic differentiation

**Fig. 6:**
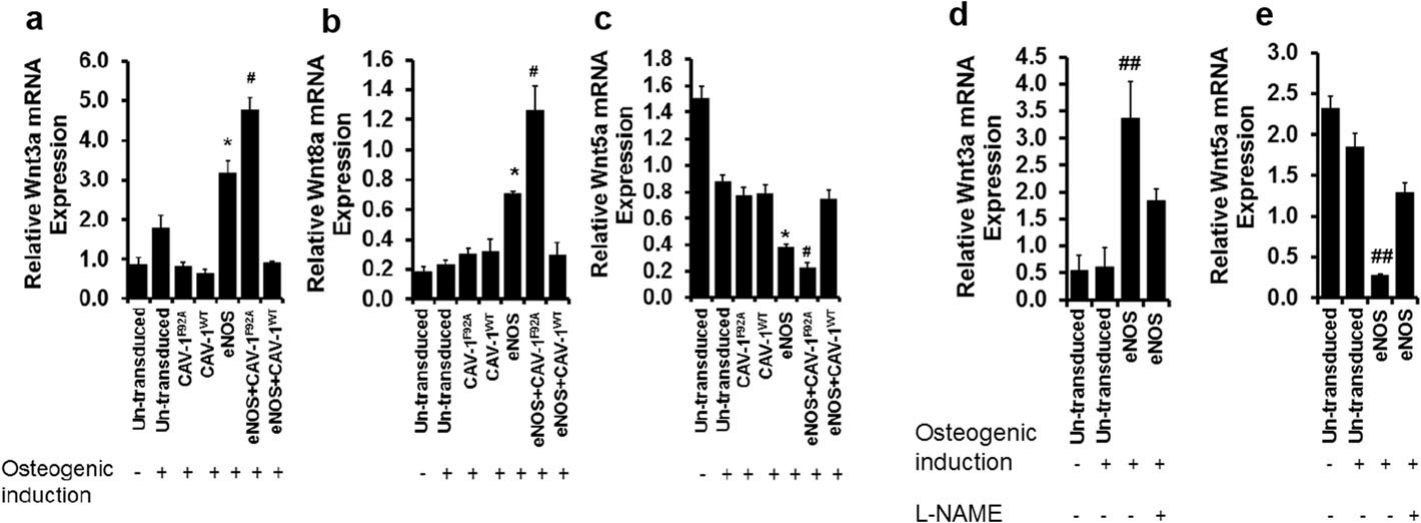
Nitric oxide signaling modulates Wnt signaling in eASCs. Relative mRNA transcript analysis by qPCR shows that endothelial nitric oxide synthase (*eNOS*) and eNOS + mutated caveolin-1 (*eNOS + CAV-1* ^*F92A*^) transduced cells increased the expression of canonical Wnt ligands **a** Wnt3a and **b** Wnt8a, whilst downregulating **c** non-canonical Wnt5a. Relative mRNA transcripts analysis by qPCR shows that treatment with 2 mM l-N^G^-nitroarginine methyl ester (*l* *-NAME*) downregulated **d** Wnt 3a and upregulated **e** Wnt5a expression. *p < 0.05  eNOS+CAV-1WT, CAV-1WT, CAV-1F92A, eASC (osteogenic induction), eASC (growth medium). and ^#^ *p* < 0.05 versus eNOS + wild-type caveolin-1 (*CAV-1* ^*WT*^), CAV-1^WT^, CAV-1^F92A^, eASC (osteogenic induction), and eASC (in DMEM); ^##^ *p* < 0.05, versus eNOS (l-NAME) eASC (osteogenic induction) and eASC (growth medium)

To examine the role of canonical and non-canonical Wnt signaling during NO-mediated osteogenic differentiation, expression of Wnt3a, Wnt8a, and Wnt5a was assessed by quantitative real-time PCR. Non-canonical Wnt5a expression was reduced in eASC^eNOS^ (Fig. 6c), and was significantly further decreased in eASC^eNOS+CAVF92A^ (Fig. 6c). However, expression of canonical Wnt ligands Wnt3a (Fig. 6a) and Wnt8a (Fig. 6b) was upregulated in eASC^eNOS^ and significantly further increased in eASC^eNOS+CAVF92A^ (Fig. 6a and b, respectively). Treatment with 2 mM l-NAME showed downregulation of Wnt3a expression (Fig. 6d) and upregulation of Wnt5a (Fig. 6e) in eASC^eNOS^, indicating that NO modulates Wnt signaling pathway in eASCs.

**Fig. 7:**
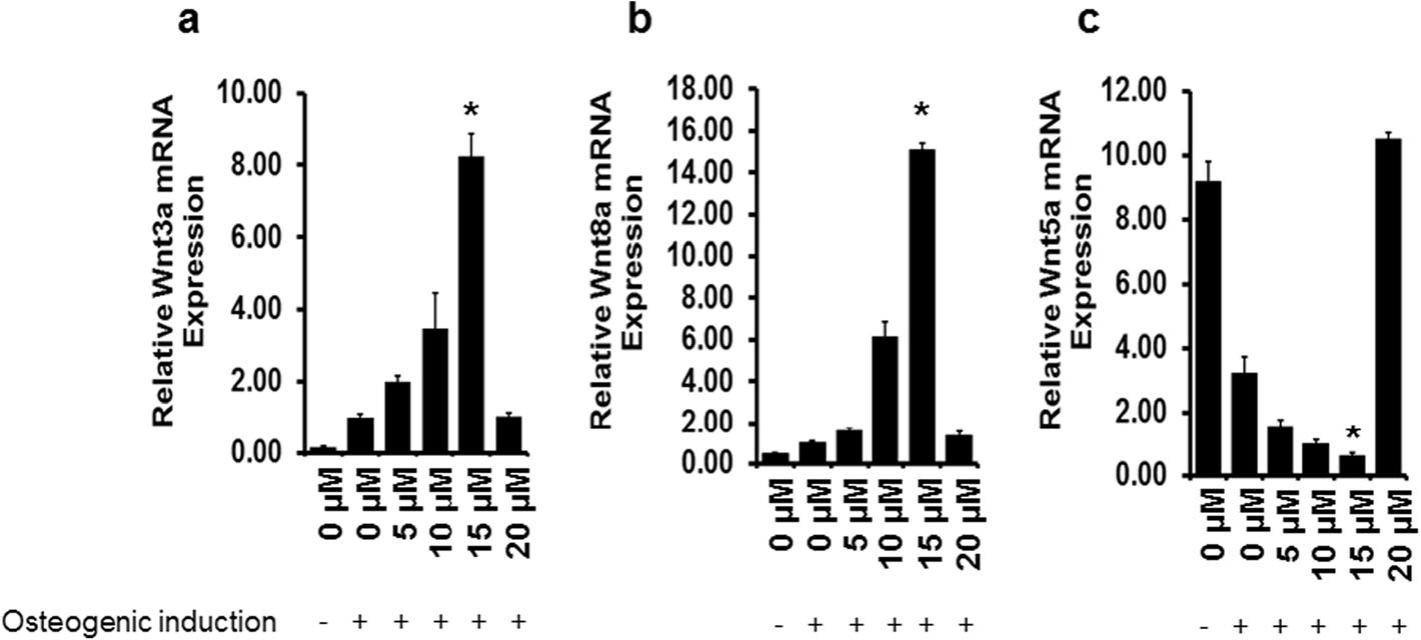
Treatment with nitric oxide donor NONOate modulates Wnt signaling. Relative mRNA transcript analysis by qPCR shows that exogenous NONOate treatment significantly upregulates the expression of canonical Wnt ligands **a** Wnt3a and **b** Wnt8a in a dose-dependent manner up to 15 μM with a corresponding downregulation of the non-canonical Wnt ligand **c** Wnt5a. *p < 0.05, versus 0 μM, 5 μM, 10 μM, 20 μM, and 0 μM (growth medium)

Furthermore, treatment with NO donor (NONOate) also resulted in increased expression of canonical Wnt ligands Wnt3a (Fig. 7a) and Wnt8a (Fig. 7b), and downregulation of non-canonical Wnt5a expression (Fig 7c) in a dose-dependent manner from 5 μM to 15 μM of NONOate. Interestingly, when the NO donor concentration was increased up to 20 μM, the effect was completely reversed by downregulating Wnt3a (Fig. 7a) and Wnt8a (Fig. 7b), and upregulating Wnt5a expression (Fig. 7c). Control cells (eASCs in normal growth medium) also showed increased expression of Wnt5a (Fig. 7c), suggesting that induction of osteogenic differentiation of eASCs requires activation of canonical Wnt signaling and suppression of non-canonical Wnt5a expression.

**Fig. 8:**
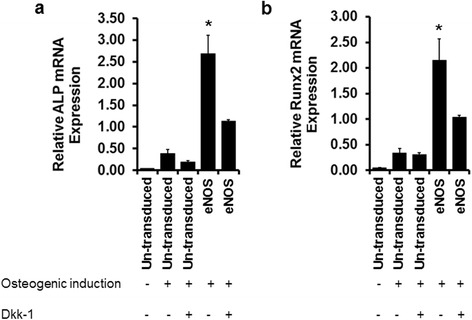
Inhibition of Wnt signaling through Dickkopf-related protein 1 (*Dkk-1*) downregulates eASC osteogenic differentiation. Relative mRNA transcript analysis by qPCR shows that Dkk-1 treatment (20 ng/mL) decreased the expression of osteoblast markers **a** alkaline phosphatase (*ALP*) and **b** Runx2 in endothelial nitric oxide synthase (*eNOS*)-transduced eASCs compared to untreated eNOS-transduced eASCs. *p < 0.05, versus eNOS (Dkk-1), eASC (osteogenic induction with Dkk-1), eASC (osteogenic induction), and eASC (growth medium)

To further analyze the relationship between NO-induced osteogenic differentiation and Wnt signaling, eNOS-transduced eASCs were treated with 20 ng/mL of the Wnt signaling inhibitor, Dickkopf-related protein 1 (Dkk-1). Dkk-1 treatment resulted in a significant downregulation of the osteoblast specific markers ALP (Fig. 8a) and Runx2 (Fig. 8b) compared to untreated eNOS transduced cells.

### Canonical Wnt3a promotes osteogenesis while non-canonical Wnt5a suppresses osteogenesis

**Fig. 9:**
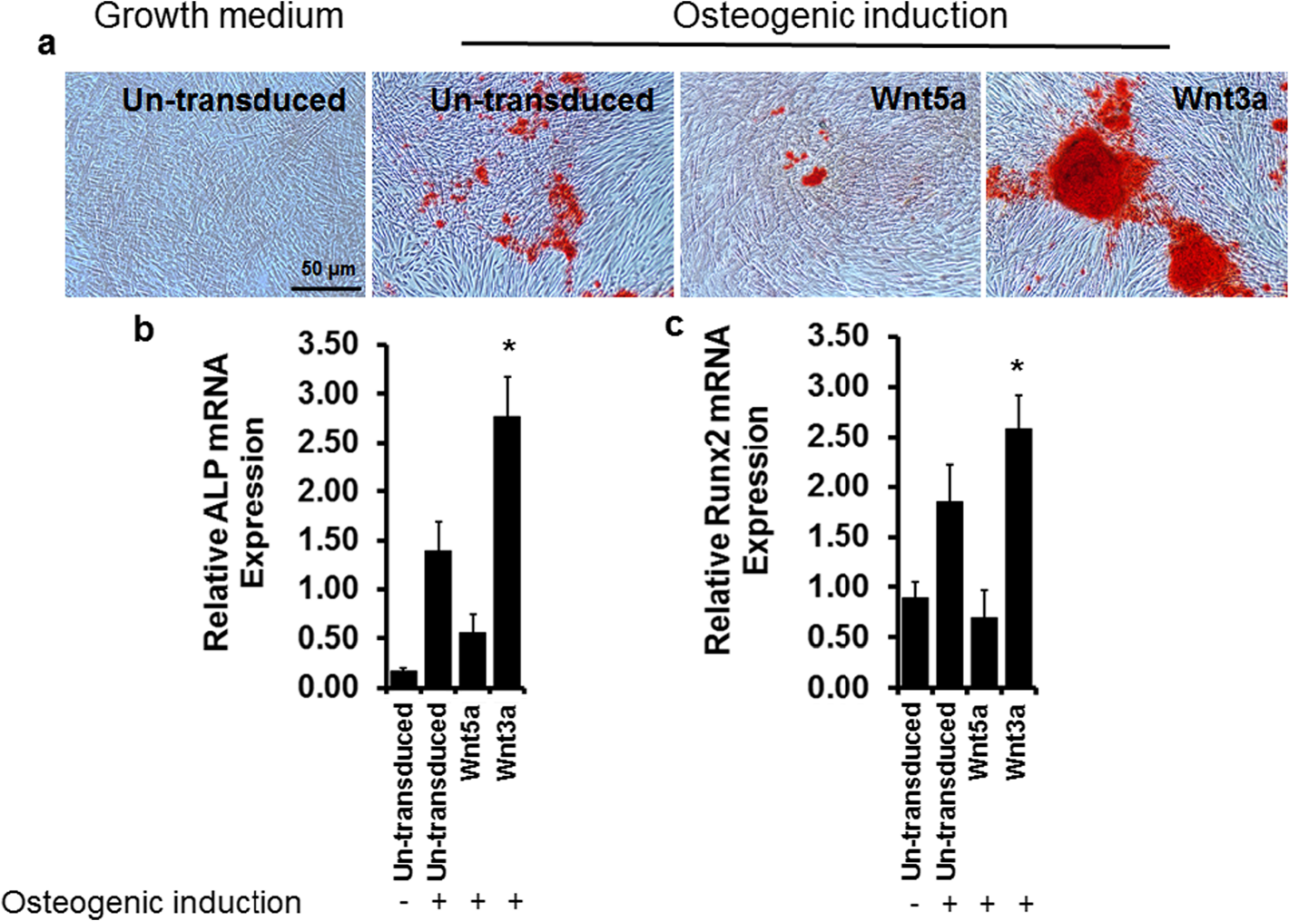
Lentiviral expression of canonical Wnt3a promotes osteogenesis and non-canonical Wnt5a results in suppressed osteogenesiss. **a** Alizarin Red S staining after 11 days in osteogenic induction medium or growth medium showing that lentiviral Wnt3a transduction increased the calcium deposition, whereas Wnt5a decreased calcium deposition levels. Relative mRNA transcript analysis by qPCR showing that Wnt3a upregulates the expression of osteoblast markers **b** alkaline phosphatase (*ALP*) and **c** Runx2 expression, whereas Wnt5a resulted in downregulation. *p < 0.05, versus Wnt5a, eASC (osteogenic induction), and eASC (growth medium)

To explore the opposite effects of canonical and non-canonical Wnt signaling pathways on eASC osteogenesis, we generated canonical Wnt3a and non-canonical Wnt5a expressing lentiviral vectors and transduced eASCs. Wnt3a-transduced eASCs (eASC^Wnt3a^) and Wnt5a-transduced eASCs (eASC^Wnt5a^) were incubated in osteogenic induction medium (OM) for 11 days. Interestingly, we found that Wnt3a (eASC^Wnt3a^) promoted osteogenesis as evident by Alizarin Red staining compared to un-transduced eASCs (eASC^WT^) (Fig. 9a). On the other hand, overexpression of Wnt5a (eASC^Wnt5a^) reduced osteogenic differentiation (Fig. 9a). Quantitative analysis of the mRNA levels by real-time PCR revealed that ALP (Fig. 9b) and Runx2 (Fig. 9c) were upregulated in the eASC^Wnt3a^ culture as compared with the ASC^WT^ culture, and downregulated in eASC^Wnt5a^ culture, suggesting that lenitiviral vector-mediated Wnt3a expression can promote osteogenesis while expression of non-canonical Wnt5a suppresses osteogenesis.

### NO promotes the canonical Wnt signaling pathway by promoting nuclear translocation of β-catenin

**Fig. 10:**
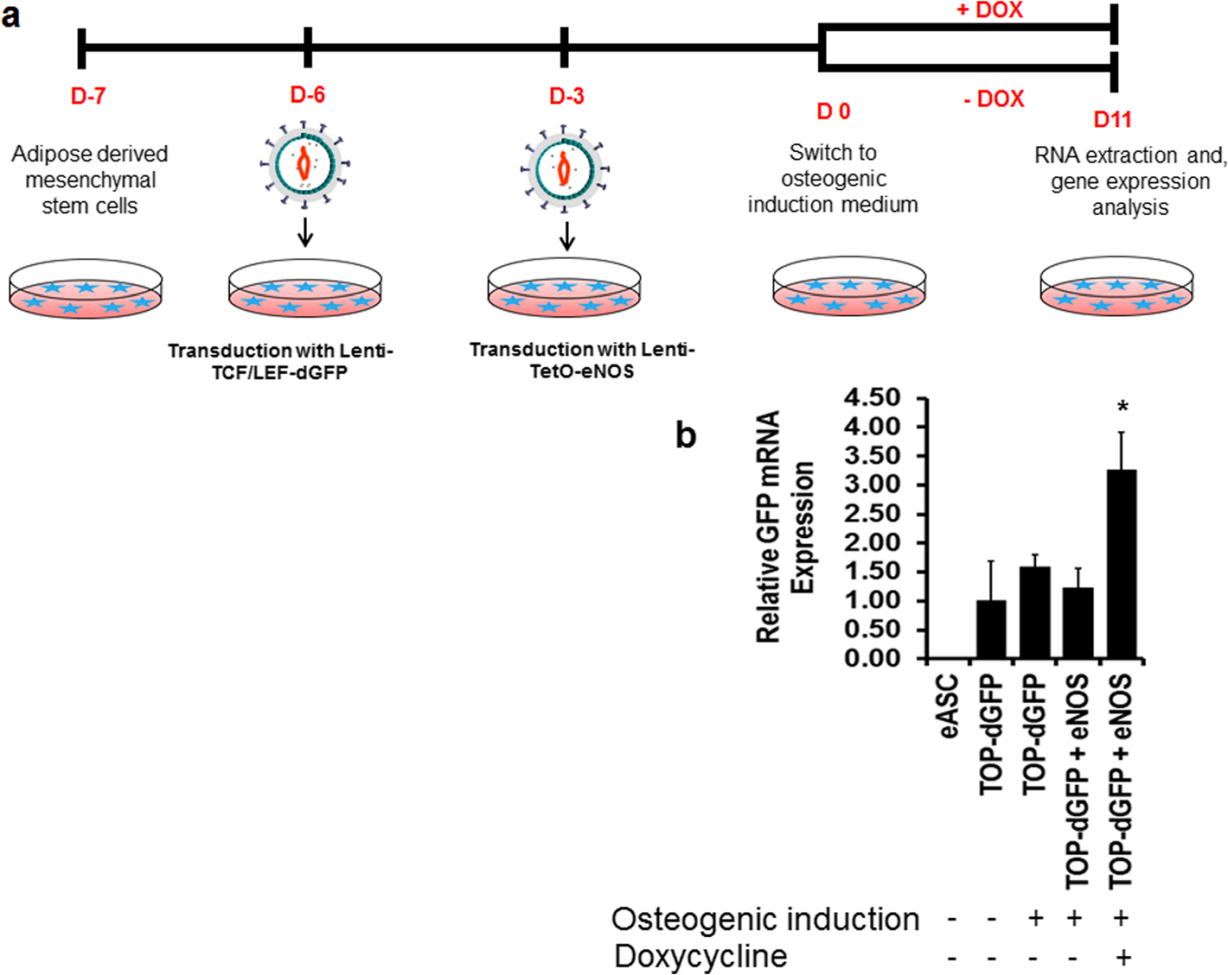
Nitric oxide signaling promotes activity of a β-catenin reporter. **a** Schematic of lentiviral transduction of a β-catenin reporter, TCF/LEF (*TOP-dGFP*), and a doxycycline (*DOX*) inducible endothelial nitric oxide synthase (*eNOS*) transduction and assessment of TCF/LEF activity during osteogenesis. **b** Relative mRNA transcript analysis by qPCR showing eNOS upregulates GFP mRNA expression when treated with DOX. *p < 0.05, versus TOP-dGFP + eNOS (no DOX), TOP-dGFP (osteogenic induction), TOP-dGFP (growth medium), and un-transduced eASC (growth medium)

To further explore mechanisms by which NO promotes canonical Wnt signaling, we used a lentiviral vector expressing GFP reporter under the control of the TCF/LEF promoter (TOPFLASH; Addgene). In the canonical Wnt signaling pathway, β-catenin translocation to the nucleus is promoted by the activation of canonical Wnt signaling [52, 53]. Accordingly, eASCs were introduced with the lentiviral TCF/LEF-dGFP reporter, and those cells were then co-transduced with doxycycline inducible eNOS expressing lentiviral vector (Fig. 10a). As a readout for nuclear translocation of β-catenin, the TCF/LEF-driven GFP mRNA expression levels were measured by quantitative real-time PCR. Under osteogenic induction conditions, increased GFP mRNA expression was demonstrated compared to non-osteogenic induction conditions (cells in growth medium) (Fig. 10b). When doxycycline was added to the OM, GFP mRNA expression was significantly increased in the eNOS-transduced cells (eASC^eNOS^) (Fig. 10b). Interestingly, when doxycycline was removed from the medium, GFP mRNA expression in eASC^eNOS^ was similar to other osteogenic induction conditions (Fig. 10b).

**Fig. 11:**
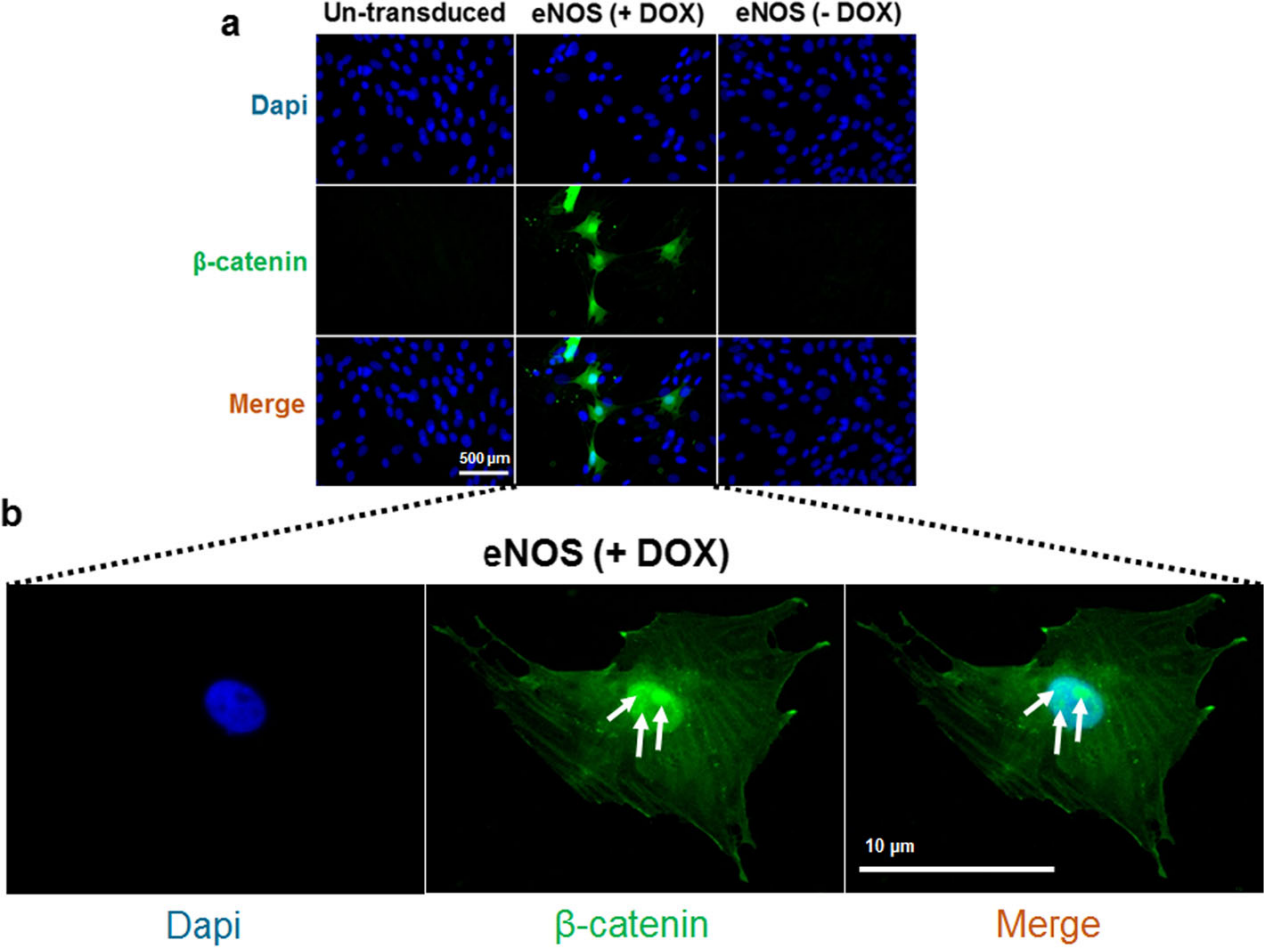
Nitric oxide promotes nuclear translocation of β-catenin. **a** Immunostaining with a β-catenin-specific monoclonal antibody reveals that the expression of β-catenin in endothelial nitric oxide synthase (*eNOS*) transduced cells when doxycycline (*DOX*) is available in the medium. **b** nuclear localization of beta catenin in eNOS transduced cells in DOX containing medium. *Arrows* indicate nuclear localisation of β-catenin

Using a β-catenin-specific monoclonal antibody, we further investigated the effect of NO on β-catenin nuclear translocation. eASCs were transduced with the doxycycline inducible eNOS lentiviral vector followed by immunostaining with β-catenin-specific monoclonal antibody (Cell Signaling Technology). When doxycycline was added to the OM, the expression of β-catenin was observed in eNOS-transduced cells (eASC^eNOS^) (Fig. 11a) in both the nucleus and cytoplasm, and when doxycycline was removed from the medium β-catenin expression in eASC^eNOS^ was reduced (Fig. 11a) to that seen in un-transduced control cells (Fig. 11a). Furthermore, we observed nuclear co-localization of β-catenin and DAPI only in doxycycline-treated eASC^eNOS^ suggesting that NO may promote nuclear localization of β-catenin (Fig. 11b). As a control for the primary antibody, immunostaining was carried out in the absence of the primary antibody specific to β-catenin (Additional file 2: Figure S2).

**Fig. 12:**
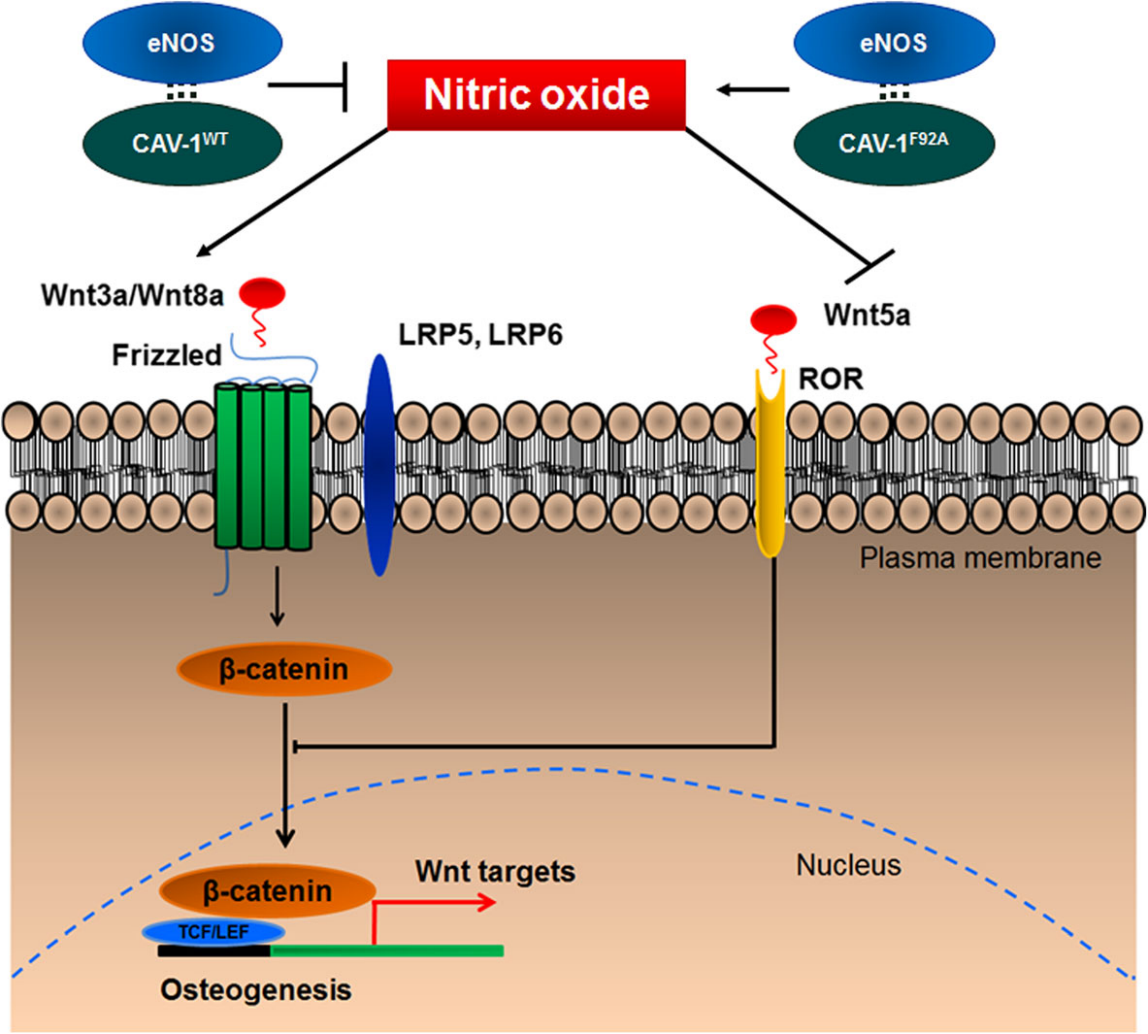
Proposed signaling mechanism underlying osteogenic differentiation induced by NO in eASCs. Molecular control of NO levels may activate and suppress the expression of endogenous canonical and non-canonical Wnt ligands, respectively, to promote nuclear localization of β-catenin and subsequent activation of osteogenic differentiation through promoting osteoblast-specific gene transcription. *CAV-1* ^*F92A*^ mutated caveolin-1, *CAV-1* ^*WT*^ wild-type caveolin-1, *eNOS* endothelial nitric oxide synthase

Together, these findings support the paradigm that cellular environments rich in bioavailable NO through either genetic modification or exogenous sources can modulate Wnt signaling, by upregulating the canonical and downregulating the non-canonical pathways resulting in increased osteogenic differentiation (Fig. 12).

## Discussion

NO plays an important role in osteogensis, bone remodeling, and metabolism [54–56]. It has been reported that both iNOS and eNOS play a role in osteogenesis of embryonic stem cells [57]. We [4] and others [58] have shown that MSCs do not express eNOS. Therefore, in order to investigate the role of eNOS in osteogenic differentiation of eASCs, in this study eASCs were genetically modified by lentiviral vector-based eNOS. ASCs are promising candidates for stem cell-based therapy for bone repair [59], and the role of eNOS-mediated NO synthesis and its downstream effect on osteogenesis of MSCs remains to be explored. We found that eNOS gene transfer by lentiviral vector promoted osteoblast-specific gene expressions (Fig. 2e and f), contributing to the matrix mineralization as visualized by Alizarin Red S staining (Fig. 2b and d). Noteworthy, this osteogenic potential of eASCs^eNOS^ was significantly abrogated by l-NAME treatment (Fig. 3), suggesting that NO derived from eNOS plays a major role in enhancing osteogenesis in eASCs.

CAV-1 is a key negative regulator of eNOS activation and thus inhibits the production of NO [41, 60] and, importantly, CAV-1 is expressed endogenously in MSCs [61]. The scaffolding domain (82-101 amino acids) of CAV-1 protein interacts with eNOS at the plasma membrane and this interaction inhibits the eNOS activation reducing NO synthesis [41]. An alanine scanning approach revealed that substitution of phenylalanine at the amino acid position 92 with alanine to produce CAV-1^F92A^ mutant restored the eNOS activation and promoted NO synthesis [41]. Thus, in order to understand the contribution of caveolin-1 on the control of NO synthesis in eASC osteogenesis, we modified eASCs by expressing CAV-1^WT^ (as a negative regulator for eNOS activation) or CAV-1^F92A^ (as a positive regulator for eNOS activation) together with eNOS. Confirming a previous observation [41], we found that co-expression of eNOS and CAV-1^F92A^ increased NO production while eNOS and CAV-1^WT^ co-expressed eASCs showed reduced NO production (Fig. 2c), suggesting that CAV-1 is an important regulator of NO production in eASCs. We further found that these controlled levels of NO synthesis regulate osteogenesis, where eNOS together with CAV-1^F92A^ resulted in increased osteogenic differentiation of eASCs.

To explore the molecular basis of NO-mediated osteogenesis, we investigated the effect of NO on Wnt signaling. Wnt signaling pathways have been shown to regulate osteoblastogenesis [62], in which canonical Wnt ligands promote osteogenesis [63, 64], and non-canonical Wnt5a can inhibit the canonical Wnt signaling [65]. In the canonical Wnt pathway, binding of canonical Wnt ligands such as Wnt3a and Wnt8a to cell surface frizzled receptors results in the nuclear translocation of β-catenin [66], which ultimately binds with the TCF/LEF region to initiate the transcription of osteogenic genes such as Runx2 [62]. On the other hand, binding of non-canonical Wnt5a ligand to the ROR2 member of the Ror-family of RTKs inhibits canonical Wnt signaling by promoting β-catenin degradation, and downregulation of β-catenin reduced osteoblast-specific gene expression [67]. Our results revealed that genetic manipulation of eASCs with eNOS and CAV-1^F92A^ (eASC^eNOS+CAV-1F92A^) increased canonical Wnt3a and Wnt8a expression, whereas eASC^eNOS+CAV-1WT^ decreased Wnt3a and Wnt8a expression (Fig. 6a and b), suggesting that NO levels may regulate Wnt ligand expression and promote osteogenesis. Confirming the role of Wnt signaling on osteogenesis, inhibition of canonical Wnt signaling through Dkk-1 treatment of eNOS-expressing cells attenuated osteogenesis as evident by downregulation of osteoblast-specific Runx2 and ALP expression (Fig. 8). On the other hand, the effect of non-canonical Wnt5a expression was completely the opposite (Fig. 6c) to the canonical Wnt3a and Wnt8a expression profiles, suggesting that molecular control of NO synthesis through eNOS/CAV-1 interaction or exogenous NO treatment (Fig. 7) results in differential regulation of Wnt ligand expression and their subsequent effect on osteogenic differentiation. Furthermore, we also found that NO modulates Wnt signaling and promotes osteogenesis when a differentiation environment is enhanced with an optimum concentration of exogenous NO (Figs. 4 and 7).

It has been shown that Wnt3a can directly promote osteogenesis [68], whilst Wnt5a plays a role in self-renewal of stem cells [69]. We further investigated the direct effect of canonical Wnt3a and non-canonical Wnt5a on eASC osteogenesis through lentiviral vector overexpression. Interestingly, corroborating our results on NO-mediated Wnt-regulated osteogenesis, Wnt3a promoted osteogenesis (Fig. 9a–c) whereas Wnt5a inhibited osteogenesis (Fig. 9a–c). It was shown that increased levels of β-catenin can promote bone formation through increasing the expression of osteoblast-specific genes [70, 71], whilst abnormal osteoblast differentiation has been observed with β-catenin knockdown [70, 72]. Thus, it is possible that Wnt3a promotes osteogenesis by increasing β-catenin stability and Wnt5a may suppress osteogenesis by degrading β-catenin. NO may regulate this mechanism by increasing Wnt3a and suppressing Wnt5a ligand availability to modulate nuclear localization of β-catenin via the canonical Wnt ligand transduction pathway. In support of this, we observed that eNOS-transduced cells promoted the expression of β-catenin and its nuclear localization (Fig. 11), and a TCF/LEF-dGFP reporter assay demonstrated responsiveness in a NO-rich cellular environment (Fig. 10b), which could be controlled through the expression of DOX-inducible eNOS.

## Conclusions

In summary, our findings provide an insight into the role of NO in promoting eASC osteogenic differentiation in a cellular environment of optimum levels of NO through interaction with Wnt signaling pathways. This may lead to the development of novel cell-based therapeutic approaches for bone repair, in particular in vitro modification of MSCs by NO to optimize the endogenous Wnt signaling pathway to promote osteogenic differentiation upon subsequent transplantation.

## Additional files

Additional file 1: Figure S1. Primary antibody controls for Fig. 1. As a control for specific primary antibody binding, immunostaining was performed in the absence of primary antibodies, with only fluorophore tagged secondary antibody present. We confirm no florescence signal was detected these controls for eNOS and CAV-1. (a) Detection of nuclear staining by Dapi and (b) immunostaining performed without mouse monoclonal anti-eNOS antibody but with an anti-mouse IgG secondary antibody conjugated with Alexa 488. (c) Detection of nuclear staining by Dapi and (d) immunostaining performed without rabbit polyclonal anti-CAV-1 antibody but with an anti-rabbit IgG secondary antibody conjugated with Alexa 555. (e) Detection of nuclear staining by Dapi and (d) fluorescence detection with no mouse monoclonal anti-eNOS antibody with an anti-mouse IgG secondary antibody conjugated with Alexa 555. (f) Detection of nuclear staining by Dapi and (g) fluorescence detection with no rabbit polyclonal anti-CAV-1 antibody with an anti-rabbit IgG secondary antibody conjugated with Alexa 488. (PPT 182 kb)
Additional file 2: Figure S2. Primary antibody controls for Fig. 11. As a control for specific β-catenin primary antibody binding, immunostaining was carried out in the absence of the primary antibody with no florescence signal detected. (a) Detection of nuclear staining by Dapi and (b) fluorescence detection without rabbit monoclonal anti-β-catenin antibody with an anti-mouse IgG secondary antibody conjugated with Alexa 488. (PPT 102 kb)

## Abbreviations

ALP: Alkaline phosphatase
CAV-1: Caveolin-1
DOX: Doxycycline
eASC: Equine adipose-derived stem cell
eGFP: Enhanced green fluorescent protein
eNOS: Endothelial nitric oxide synthase
FBS: Fetal bovine serum
iNOS: Cytokine-inducible nitric oxide synthase
l-NAME: l-N^G^-nitroarginine methyl ester
MSC: Mesenchymal stem cell
NO: Nitric oxide
NOS: Nitric oxide synthase
OM: Osteogenic induction medium
PBS: Phosphate-buffered saline
PCR: Polymerase chain reaction
RT-qPCR: Reverse transcription quantitative polymerase chain reaction
