# Supplementary material for: Molecular control of nitric oxide synthesis through eNOS and caveolin-1 interaction regulates osteogenic differentiation of adipose-derived stem cells by modulation of Wnt/β-catenin signaling
Source: Stem Cell Res Ther. 2016 Dec 7;7:182. doi: 10.1186/s13287-016-0442-9 (PMC5142348; doi:10.1186/s13287-016-0442-9)
Supplement: Additional file 1: Figure S1. — Primary antibody controls for Fig. 1. As a control for specific primary antibody binding, immunostaining was performed in the absence of primary antibodies, with only fluorophore tagged secondary antibody present. We confirm no florescence signal was detected these controls for eNOS and CAV-1. (a) Detection of nuclear staining by Dapi and (b) immunostaining performed without mouse monoclonal anti-eNOS antibody but with an anti-mouse IgG secondary antibody conjugated with Alexa 488. (c) Detection of nuclear staining by Dapi and (d) immunostaining performed without rabbit polyclonal anti-CAV-1 antibody but with an anti-rabbit IgG secondary antibody conjugated with Alexa 555. (e) Detection of nuclear staining by Dapi and (d) fluorescence detection with no mouse monoclonal anti-eNOS antibody with an anti-mouse IgG secondary antibody conjugated with Alexa 555. (f) Detection of nuclear staining by Dapi and (g) fluorescence detection with no rabbit polyclonal anti-CAV-1 antibody with an anti-rabbit IgG secondary antibody conjugated with Alexa 488. (PPT 182 kb) [file 13287_2016_442_MOESM1_ESM.ppt]

## Slide 1
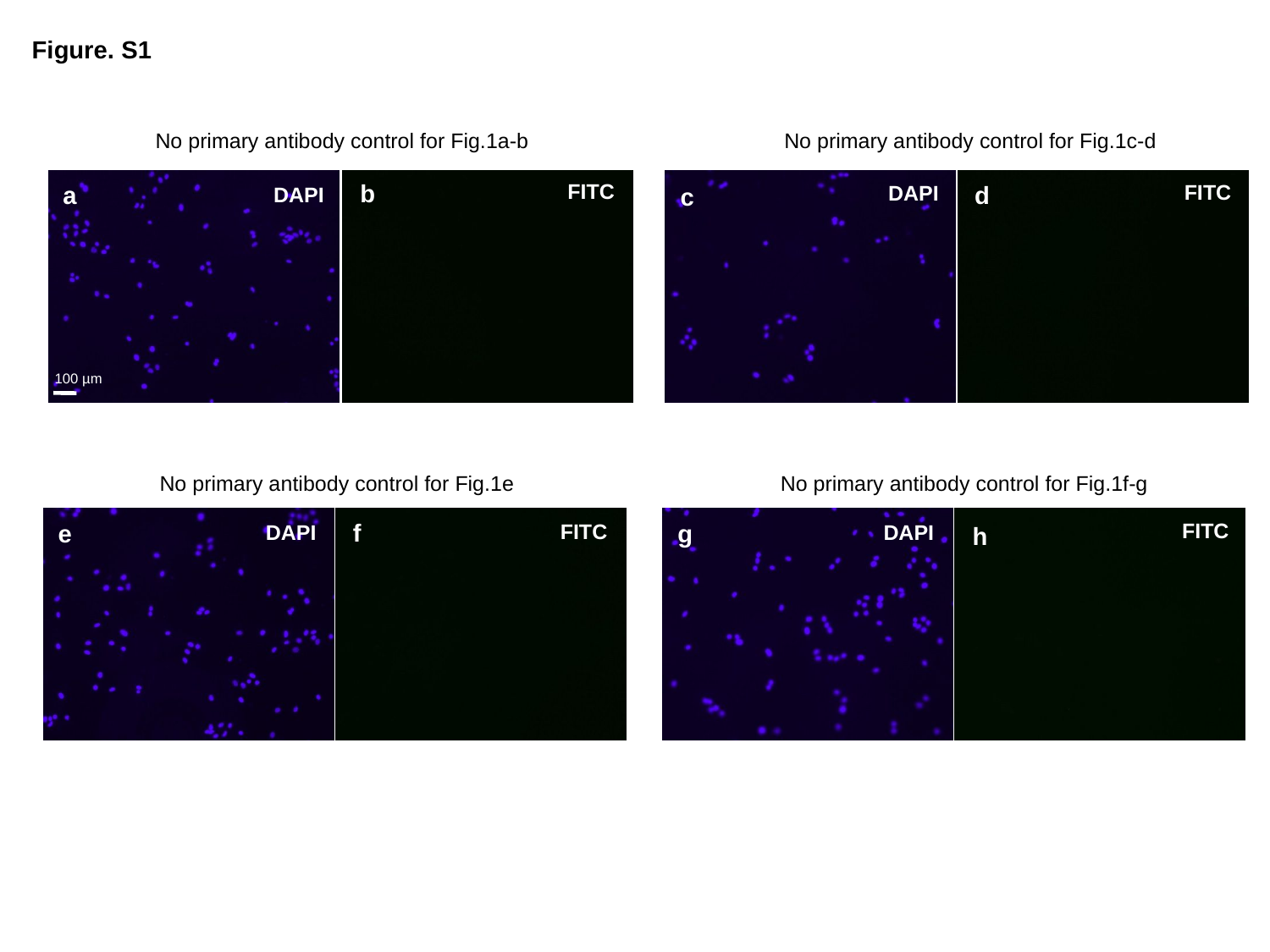

Figure. S1
No primary antibody control for Fig.1a-b
No primary antibody control for Fig.1c-d
FITC
b
d
FITC
a
DAPI
DAPI
c
100 µm
No primary antibody control for Fig.1e
No primary antibody control for Fig.1f-g
f
FITC
g
e
FITC
DAPI
DAPI
h
