# Supplementary figures and images for: Molecular control of nitric oxide synthesis through eNOS and caveolin-1 interaction regulates osteogenic differentiation of adipose-derived stem cells by modulation of Wnt/β-catenin signaling
Source: Stem Cell Res Ther. 2016 Dec 7;7:182. doi: 10.1186/s13287-016-0442-9 (PMC5142348; doi:10.1186/s13287-016-0442-9)

## Slide 1
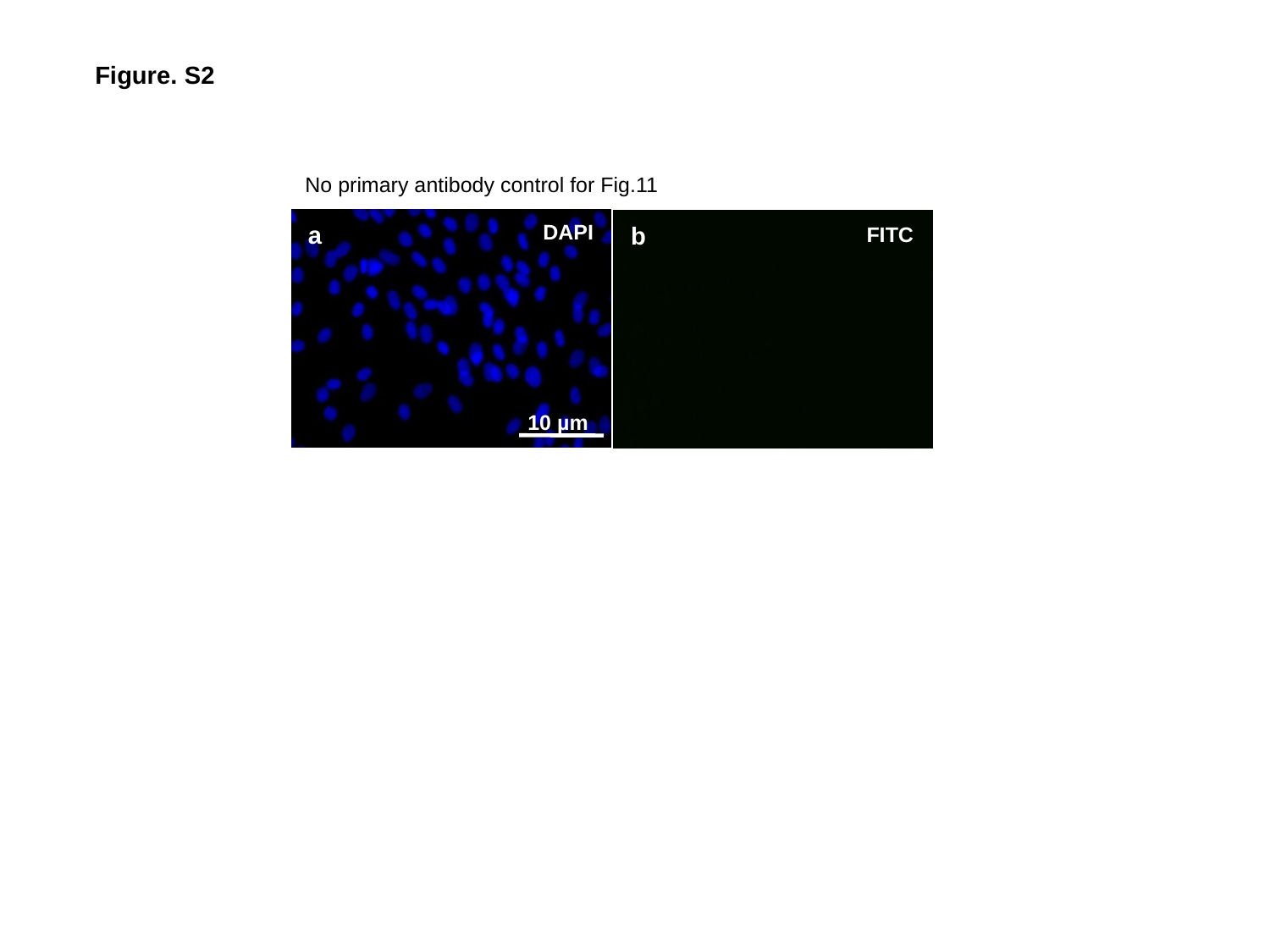

Figure. S2
No primary antibody control for Fig.11
DAPI
a
b
FITC
10 µm

Supplement: Additional file 2: Figure S2. — Primary antibody controls for Fig. 11. As a control for specific β-catenin primary antibody binding, immunostaining was carried out in the absence of the primary antibody with no florescence signal detected. (a) Detection of nuclear staining by Dapi and (b) fluorescence detection without rabbit monoclonal anti-β-catenin antibody with an anti-mouse IgG secondary antibody conjugated with Alexa 488. (PPT 102 kb) [file 13287_2016_442_MOESM2_ESM.ppt]
